# *Wohlfahrtiimonas chitiniclastica* Bacteremia in Homeless Woman

**DOI:** 10.3201/eid1506.080232

**Published:** 2009-06

**Authors:** Stanislas Rebaudet, Séverine Genot, Aurélie Renvoise, Pierre-Edouard Fournier, Andreas Stein

**Affiliations:** Hôpital Universitaire de la Conception, Marseille, France (S. Rebaudet, S. Genot, A. Stein); Unité des Rickettsies, Marseille (A. Renvoise, P.-E. Fournier, A. Stein)

**Keywords:** Wohlfahrtiimonas, Ignatzschineria, Schineria, homeless, myiasis, lice, ectoparasitism, parasites, France, letter

**To the Editor:** In May 2006, a 60-year-old homeless woman with a history of alcoholism was admitted to the emergency department of the Conception Hospital, Marseille, France. Firefighters had just found her in an abandoned container in the outskirts of the city, beside the body of her companion, who had died several days earlier. She described no symptoms other than fatigue. On examination, she was found to be dirty and covered with thousands of body and hair lice; dozens of insect larvae were in her hair. She was mildly febrile (38°C) and had widespread excoriations but no sign of localized bacterial infection. Head shaving exposed superficial ulcers on her scalp but no maggots. Blood analysis showed marked neutropenia (0.44 ×10^9^/L), thrombocytopenia (28 × 10^9^/L), a marked but well-tolerated iron deficiency anemia (hemoglobin 6.8g/dL), and a C-reactive protein level of 182 mg/L. Louse infestation was treated with a single dose of ivermectin (12 mg), and the woman was hospitalized. On day 3, she was still febrile. Louse-borne borreliosis had been ruled out by a negative blood smear, and results of serologic testing and molecular screening of lice for the other 2 louse-transmitted bacteria, *Rickettsia prowazekii* and *Bartonella quintana* (*1*), were negative.

In contrast, 2 cultures of blood taken at the time of admission grew gram-negative rods susceptible to amoxicillin, ceftriaxone, imipenem, ciprofloxacin, amikacin, and trimethoprim/sulfamethoxazole. However, phenotypic tests failed to identify this bacterium with accuracy. Intravenous therapy with ceftriaxone at 2 g/d was initiated, and the patient’s fever, neutropenia, and thrombocytopenia improved. Scalp wounds healed with local care. Using 16S rRNA gene amplification and sequencing as previously described (*2*), we identified the bacilli as *Wohlfahrtiimonas chitiniclastica* and determined its similarity to be 99.5% with strain E43 (GenBank accession no. AJ517825). The 16S rRNA sequence obtained from the patient’s strain was deposited in GenBank under no. EU484335. The strain was deposited in the Collection de Souches de l’Unité des Rickettsies (CSUR; World Data Center for Microorganisms 875, http://ifr48.timone.univ-mrs.fr/portail2/index.php?option = com_content&task = view&id = 96&Itemid = 52) under reference CSUR P16.

*W. chitiniclastica* is a recently described γ-proteobacterium isolated from larvae of the parasitic fly *Wohlfahrtia magnifica* (*3*). Although the pathogenicity of this new species for humans is as yet undescribed, it is phylogenetically close to *Ignatzschineria larvae*, another bacterium associated with *W. magnifica* larvae (*4*), which cause severe wound myiasis in cattle (*5*). Because of its strong chitinase activity, *I. larvae* may play a role in the metamorphosis of its host fly, as has been observed for other fly symbionts, and thus may be a symbiont of *W. magnifica* flies (*6*). The bacterium was later discovered in swine waste in Quebec (*7*). In 2007, three publications renewed researchers’ interest in *I. larvae*. First it was reclassified as the only species within the genus *Ignatzschineria* (*4*). Then 2 case reports demonstrated that it plays a role as a human pathogen (*8*,*9*). Both described an *I. larvae* bacteremia in adults with myiasis in southeastern France. The first patient was an elderly farmer with diabetes and myiasis of the leg, scrotum, and anus (*8*). The second patient was a middle-aged homeless man with a history of alcoholism who also had foot wound myiasis (*9*).

We report *W. chitiniclastica* bacteremia also in a homeless woman from southeastern France. Although we did not test body lice for *W. chitiniclastica*, we believe that the bacteremia originated from the patient’s scalp maggots. Unfortunately, as previously reported for cases of *I. larvae* bacteremia, the maggots had been rapidly discarded, permitting neither bacterial analysis nor entomologic identification. However, these larvae may have been from *W. magnifica* flies. These flies are present in southern France, and although they are not typically found at low altitude and in a semiurban environment, their distribution is known to be progressively expanding, in part because of their broad adaptation capacities. Animal hosts for *W. magnifica* flies are numerous, but humans can also be infected; >10 cases of this myiasis in humans have been reported in Europe, Asia, Morocco, and Egypt. The scalp was affected in 2 of these patients (*10*).

Among homeless persons, ectoparasitism is very common; body lice (*Pediculus humanus humanus*) are of particular interest because they transmit 3 bacterial bloodstream infections: trench fever (*B. quintana*), epidemic typhus (*R. prowazekii*), and louse-borne relapsing fever (*Borrelia recurrentis*) (*1*). Myiasis should also be considered as a relevant type of ectoparasitism in homeless and hygiene-deficient persons. In addition, like body lice, ticks, and fleas, fly larvae should also be regarded as another potential source of specific arthropod-borne bacterial systemic infections.
